# Fatty acid–related immune network in psoriasis: metabolic regulation of innate and adaptive immunity

**DOI:** 10.3389/fphar.2026.1731683

**Published:** 2026-03-05

**Authors:** Pengfei Wen, Xiaoxue Zhuo, Siliang Xue

**Affiliations:** Department of Dermatovenerology, West China Hospital, Sichuan University, Chengdu, China

**Keywords:** fatty acid oxidation, immunomodulation, peroxisome proliferator-activated receptor γ, psoriasis, short-chain fatty acids, Treg/Th17 axis

## Abstract

Psoriasis is a chronic inflammatory skin disorder driven by dysregulation of the Treg/Th17 axis, where enhanced Th17 activity promotes keratinocyte proliferation and inflammation, while impaired Treg function exacerbates immune dysregulation. Emerging evidence highlights peroxisome proliferator-activated receptor γ (PPARγ) as a key regulator of fatty acid oxidation (FAO), a metabolic pathway critical for Treg differentiation and function. PPARγ activation enhances FAO via upregulation of CD36, CPT1, and AMPK signaling, while suppressing glycolysis, thereby skewing the Treg/Th17 balance toward immune tolerance. Concurrently, short-chain fatty acids (SCFAs), microbial metabolites with immunomodulatory properties. ameliorate psoriatic inflammation by promoting Treg expansion, inhibiting Th17 polarization, and modulating innate immune cells (neutrophils, dendritic cells, and macrophages). SCFAs exert their effects through receptor-dependent signaling and epigenetic mechanisms (HDAC inhibition), while derivative compounds and probiotic interventions enhance therapeutic potential. This review summarizes mechanistic insights into PPARγ-driven FAO and SCFA-mediated immunomodulation, proposing novel metabolic and microbiome-targeted strategies for psoriasis treatment.

## 1 Introduction

Psoriasis is a chronic inflammatory skin disease affecting the global population, clinically marked by erythematous, scaly plaques (Xiong et al., 2025). Central to psoriasis pathogenesis is the dysregulated regulatory T (Treg) and Th17 axis. Th17 cells secrete IL-17 and IL-22, promoting keratinocyte proliferation and amplifying inflammation, whereas Tregs suppress immune activation through IL-10 and TGF-β (Kim et al., 2022; Wu et al., 2023). Upregulation of FAO supports Treg differentiation and inhibits glycolytic flux, thereby limiting Th17 cell development and favoring immune tolerance (Wang et al., 2022; Lu et al., 2025; Chen et al., 2021; Sarandi et al., 2024; Sivasami et al., 2023). Peroxisome proliferator-activated receptor γ (PPARγ), a nuclear receptor, orchestrates FAO by enhancing mitochondrial fatty acid uptake and β-oxidation, boosting Treg metabolic fitness and function (Miao et al., 2022; Sobolev et al., 2022). By simultaneously repressing glycolysis, PPARγ activation rebalances the Treg/Th17 axis and mitigates psoriatic inflammation.

In parallel, short-chain fatty acids (SCFAs)—microbial metabolites such as acetate, propionate, and butyrate—exert potent immunomodulatory effects via histone deacetylase (HDAC) inhibition and G-protein–coupled receptor signaling (Liu et al., 2023; Briganti et al., 2024). SCFAs not only promote Treg differentiation and suppress Th17 polarization but also modulate innate immune cells, including neutrophils, dendritic cells, and macrophages, contributing to the resolution of cutaneous inflammation (Liu et al., 2023; Du et al., 2024). Importantly, SCFAs activate PPARγ and converge on shared metabolic and transcriptional pathways, linking host lipid metabolism and gut microbiota–derived immunoregulation (Oh et al., 2019; Nepelska et al., 2017). This metabolic regulation offers a unifying mechanistic framework through which adaptive and innate immune responses are coordinated in psoriasis (Komine, 2020; Zhang et al., 2024; Sarandi et al., 2023). By integrating emerging evidence on PPARγ-mediated FAO and SCFA signaling, this review proposes a comprehensive model of immune-metabolic reprogramming in psoriasis and highlights therapeutic strategies targeting lipid metabolism and the microbiome to restore immune equilibrium and attenuate disease severity.

## 2 The role of PPARγ in FAO

Peroxisome proliferator-activated receptors (PPARs), including PPARα, PPARγ, and PPARβ/δ, are nuclear receptors regulating metabolism and immunity. PPARγ, a key isoform, modulates FAO, immune tolerance, and inflammation (Sobolev et al., 2022; Varga et al., 2011; Kim et al., 2023). In skin, it maintains immune homeostasis, controls proliferation/differentiation, and regulates inflammation, potentially influencing psoriasis via FAO modulation (Briganti et al., 2024). FAO aerobically catabolizes fatty acids into CO_2_ and H_2_O, involving uptake, activation, mitochondrial translocation (via CPT1/2), β-oxidation, and TCA cycle oxidation. PPARγ upregulates FAO by enhancing acyl-CoA synthesis, mitochondrial import, and Treg cell differentiation (Zeng et al., 2023; Tufail et al., 2024). Mechanistically, the FAO process in Treg cells proceeds through four interconnected stages: Lipid Uptake: PPARγ activation induces the expression of fatty acid transporters such as CD36 and FATPs. CD36 facilitates the uptake of long-chain fatty acids across the plasma membrane, a prerequisite for initiating FAO (Miao et al., 2022; Liu et al., 2021). FATP1, also upregulated by PPARγ, assists in intracellular transport and activation of fatty acids (Matsumoto et al., 2019; Nakamura et al., 2014). Mitochondrial Import: Once internalized, fatty acids are converted to acyl-CoA derivatives and transported into mitochondria via carnitine palmitoyltransferase 1 (CPT1), the rate-limiting enzyme in FAO. PPARγ, in conjunction with PGC-1α, enhances CPT1 expression, ensuring efficient mitochondrial import (Yamane et al., 2011; Du et al., 2019). β-Oxidation and Energy Generation: Within mitochondria, acyl-CoAs undergo β-oxidation to generate acetyl-CoA, NADH, and FADH_2_. PGC-1α co-activates FAO enzymes and supports mitochondrial biogenesis, while AMPK activation augments this process by directly phosphorylating and activating CPT1A, thereby increasing β-oxidation flux (Li et al., 2025; Abu et al., 2023; Shi et al., 2025; Chen et al., 2025). Treg Stabilization and Differentiation: The elevated β-oxidation flux sustains Treg cell metabolism, upregulates Foxp3 expression, and promotes secretion of IL-10 and TGF-β. Acetyl-CoA derived from FAO facilitates histone acetylation (H3K27ac), reinforcing Treg lineage commitment (Zhang et al., 2022; Kanda et al., 2021; Pompura et al., 2021). Simultaneously, increased FAO antagonizes glycolysis, limiting Th17 polarization and restoring immune homeostasis in psoriasis (Sarandi et al., 2023; Eleftheriadis et al., 2016; Cluxton et al., 2019). These integrated steps demonstrate that PPARγ orchestrates a coordinated FAO program—initiating at lipid uptake and culminating in Treg stabilization—through the concerted action of CD36, FATPs, CPT1, PGC-1α, and AMPK.

## 3 PPARγ regulates the T Cell via promotion of FAO

### 3.1 FAO preferentially fuels Treg cells

Dysregulation of the Treg/Th17 axis constitutes a central pathogenic mechanism in psoriasis. Treg cells maintain immune tolerance via TGF-β and IL-10 secretion, sustaining cutaneous homeostasis. In contrast, Th17 cells drive inflammation by releasing IL-17 and IL-21, promoting keratinocyte proliferation, inhibiting apoptosis, and amplifying the inflammatory cascade (Zhang et al., 2010). The expansion of Th17 cells alongside Treg depletion exacerbates psoriatic pathology. Restoration of Treg/Th17 balance through metabolic modulation—specifically by enhancing fatty acid oxidation (FAO)—offers therapeutic potential. Tregs rely on FAO for energy and function, with exogenous fatty acids supporting their proliferation and survival. FAO upregulates Foxp3 expression, facilitating Treg differentiation from naïve CD4^+^ T cells. CD36-mediated long-chain fatty acid uptake augments FAO and Foxp3 induction, whereas CD36 inhibition impairs Foxp3 expression in palmitate-stimulated T cells (Wang H. et al., 2020; Sun et al., 2024). AMP-activated protein kinase (AMPK) activation phosphorylates CPT1A, boosting mitochondrial FAO and reinforcing Foxp3-driven Treg lineage commitment (Yuan et al., 2013; Lee et al., 2015; Saadh et al., 2023). Moreover, FAO enhances the anti-inflammatory cytokine output of Tregs, including IL-10 and TGF-β (Hao et al., 2024). Collectively, these findings underscore FAO as a key metabolic regulator of Treg homeostasis and immunosuppressive function in psoriasis, suggesting that therapeutic strategies targeting FAO and the AMPK–CPT1A axis may mitigate inflammatory responses and reestablish immune equilibrium.

### 3.2 PPARγ promotes Treg differentiation by enhancing FAO

Unlike other T cell subsets, Treg cells depend predominantly on FAO for their energy requirements. Agonists of PPARγ have been shown to facilitate the differentiation of naïve T cells into Tregs (Lim et al., 2021), suggesting that PPARγ activation promotes FAO, which in turn supports Treg lineage commitment and has therapeutic potential in psoriasis. PPARγ activation enhances Treg responses via upregulation of CD36/CPT1-mediated FAO pathways (Miao et al., 2022). Beyond promoting Treg differentiation, PPARγ also enhances Foxp3 expression and the secretion of IL-10 and TGF-β through FAO upregulation (Yero et al., 2023). These transcriptional and cytokine responses have demonstrated efficacy in suppressing psoriatic inflammation, underscoring the role of PPARγ in fostering Treg development and function to alleviate disease. The enhancement of Foxp3 expression and suppression of RORγt by PPARγ is primarily mediated through FAO-dependent metabolic reprogramming and downstream epigenetic regulation (Cluxton et al., 2019; Klotz et al., 2009). Specifically, PPARγ activation upregulates CPT1A and CD36, increasing mitochondrial fatty acid uptake and β-oxidation, thereby elevating intracellular acetyl-CoA levels, a key metabolite that promotes histone acetylation. This process enhances H3K27 acetylation at the *Foxp3* promoter, facilitating its transcriptional activation (Zhang et al., 2022; Hao et al., 2021). Concurrently, increased FAO flux inhibits glycolytic metabolism, which is known to drive *Rorc* expression and Th17 differentiation. Moreover, activation of AMPK by PPARγ agonists further amplifies this metabolic shift by phosphorylating and activating CPT1A, reinforcing Treg lineage commitment (Sobolev et al., 2022; Chang et al., 2024; Kanno et al., 2025; Wang et al., 2025). Despite these FAO-linked mechanisms are experimentally supported, additional contributions from chromatin remodeling complexes or PPARγ-mediated cytokine modulation, such as suppression of IL-6 and IL-23, remain plausible and merit further investigation.

### 3.3 FAO suppresses glycolysis and Th17 cell differentiation

**FIGURE 1:**
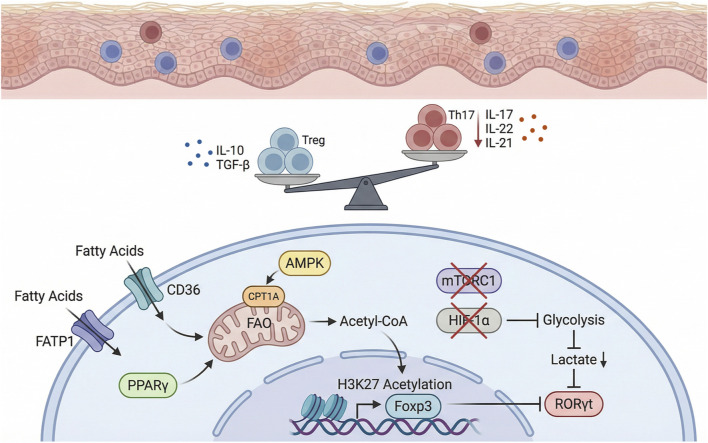
PPARγ-mediated fatty acid metabolism-immune crosstalk in psoriasis.

FAO and glycolysis exert opposing regulatory effects on Treg and Th17 cell differentiation. FAO promotes Foxp3 expression and enhances IL-10 and TGF-β production in Treg cells, while glycolysis drives RORγt expression and increases IL-17/IL-23 production in Th17 cells (Sarandi et al., 2023; Shi et al., 2025; Cluxton et al., 2019). Treg cells exhibit greater lipid oxidation and reduced glycolytic flux compared to effector CD4^+^ T cells, indicating that enhanced FAO inhibits Th17 polarization and favors Treg expansion (Michalek et al., 2011). FAO increases intracellular acetyl-CoA, promoting histone acetylation at the Foxp3 locus and facilitating Treg commitment. Conversely, glycolysis leads to lactate accumulation, redox imbalance, and NAD^+^ depletion, fostering RORγt-driven Th17 differentiation (Lu et al., 2021; Shi and Chi, 2019). In psoriasis, glycolysis-derived lactate enhances IL-17 production by γδT cells, and inhibition of lactate metabolism or RORγt reduces skin inflammation (Subudhi et al., 2024; Smith et al., 2016; Kao et al., 2025). Therefore, boosting FAO while suppressing glycolysis downregulates Th17 transcription and inflammatory cytokine production, ultimately ameliorating psoriasis-like inflammation (Sarandi et al., 2023). In psoriatic arthritis models, AMPK activation reduces Th17-driven inflammation (Yu et al., 2025), while clinical studies show diminished AMPK activity and heightened mTOR signaling in psoriatic lesions and PBMCs (Yan et al., 2018). Moreover, mTORC1–HIF-1α signaling enhances Rorc transcription and glycolytic gene expression, sustaining Th17 pro-inflammatory phenotypes (Dang et al., 2011; Sun et al., 2017). PPARγ alleviates psoriatic inflammation primarily through metabolic reprogramming—enhancing FAO and repressing glycolysis—to restore the Treg/Th17 axis. Kim et al. (2015) reported that PPARγ agonists inhibit Th17 differentiation and reduce IL-17 and IL-22 production by promoting FAO. PPARγ also suppresses Th17 transcription by downregulating RorγT via FAO enhancement (Klotz et al., 2009). Conversely, PPARγ-deficient mice exhibit significantly elevated IL-17A and IL-17F mRNA levels in CD4^+^ T lymphocytes compared to wild-type controls (Liu et al., 2016). Similarly, in colonic tissues from inflammatory bowel disease mouse models, PPARγ deficiency in macrophages leads to upregulation of IL-22 (Hontecillas et al., 2011). These studies collectively indicate that PPARγ activation downregulates Th17-associated cytokines IL-17 and IL-22, whereas PPARγ deficiency enhances their expression. Since these cytokines are well-established drivers of psoriatic inflammation, PPARγ-mediated FAO enhancement plays a crucial role in reducing Th17 activity and its associated inflammatory cytokine profile (Figure 1).

## 4 Potential mechanisms of SCFAs in psoriasis treatment

### 4.1 Cellular entry pathways of SCFAs

SCFAs enter cells via passive diffusion, carrier-mediated transport, and receptor activation, each modulating immune responses. Passive diffusion allows SCFAs to cross intestinal epithelia into circulation. Carrier proteins include sodium-coupled monocarboxylate transporter 1 (SMCT1/SLC5A8), which co-transports SCFAs (propionate, butyrate) with Na^+^, influencing indoleamine 2,3-dioxygenase 1 (IDO1) and aldehyde dehydrogenase 1A2 (ALDH1A2) in dendritic cells (DCs), promoting regulatory T cell (Treg) differentiation (Sivaprakasam et al., 2017). Monocarboxylate transporter 1 (MCT1/SLC16A1) facilitates pH-dependent SCFA transport (e.g., acetate, propionate) without Na^+^ coupling (Pfannkuche et al., 2014). Additionally, SCFAs activate receptors like GPR41, GPR43, GPR109A, Olfr78, OR51E2, aryl hydrocarbon receptor (AHR), and PPARγ (Chang et al., 2024). Receptor binding triggers signaling cascades regulating inflammation and immunity. Recent studies show SCFAs modulate cytokine production in neutrophils, macrophages, DCs, and T cells, aiding inflammation resolution (Yao et al., 2022; Xiao et al., 2022). These mechanisms highlight SCFAs’ immunomodulatory potential and therapeutic relevance in psoriasis (Xiao et al., 2022; Simancas-Racines et al., 2025; Kim et al., 2014).

### 4.2 SCFAs regulate Th17/Treg balance to modulate psoriasis pathogenesis

Psoriasis pathogenesis is governed by the TNF-α/IL-23/IL-17 axis and dysregulated keratinocyte differentiation (Takeshita et al., 2017; Sieminska et al., 2024; Menter et al., 2021; Chiricozzi et al., 2011). IL-23 not only sustains the expansion of Th17 cells but also facilitates the phenotypic reprogramming of regulatory T cells (Tregs) into Th17-like cells, while IL-17A suppresses TGF-β1 and Foxp3 expression, thereby compromising Treg-mediated immune regulation (Kanda et al., 2021; Bovenschen et al., 2011). Tregs exert their immunosuppressive functions through direct cell–cell interactions, the secretion of anti-inflammatory cytokines (IL-10, TGF-β, IL-35), and cytolytic mechanisms (Pietraforte and Frasca, 2023). SCFAs, particularly butyrate, have emerged as pivotal metabolic mediators in Treg biology. Butyrate engages GPR41 receptors on thymic epithelial cells to induce AIRE expression, thereby promoting the development of thymus-derived Tregs (Kanda et al., 2021). SCFAs also enter T cells to inhibit HDACs, enhancing H3 acetylation at the Foxp3 locus while suppressing accessibility at Rorc and Il17a enhancers, thereby promoting peripheral Treg (pTreg) differentiation and repressing Th17 lineage commitment (Martin-Gallausiaux et al., 2018). In contrast, SCFA-induced HDAC inhibition may also epigenetically repress Th17-associated loci, such as IL-17A and Rorc, through a reduction in histone acetylation or modulation of chromatin accessibility at these pro-inflammatory gene sites (Lai et al., 2023; McBride et al., 2023; Luu et al., 2019). This dual mechanism—enhanced histone acetylation at the Foxp3 promoter and suppressed accessibility at Rorc—supports a transcriptional shift favoring Treg lineage commitment over Th17 differentiation (Kanda et al., 2021; Nussbaum et al., 2021; Guendisch et al., 2017). Recent studies have demonstrated that butyrate-mediated HDAC inhibition reduces RORγt expression and IL-17A production in Th17-skewed CD4^+^ T cells, likely through downregulation of H3K27 acetylation at Th17 gene enhancers (McBride et al., 2023; Zhou et al., 2020). In addition to CD4^+^ T lymphocytes, cytotoxic CD8^+^ T cells also secrete a range of cytokines, including IL-2, IFN-γ, TNF-α, IL-17, and members of the IL-22 cytokine family, each playing a key role in psoriasis’s development (Nedoszytko et al., 2014; Ovigne et al., 2001). Under certain conditions, SCFAs suppress the production of pro-inflammatory cytokines such as IL-6 and IL-23, which are involved in the activation of the RAR-related orphan receptor γt (RORγt) and Th17 cell differentiation. By dampening the expression of these cytokines, SCFAs reduce Th17 cell frequency (Asarat et al., 2016). Thus, modulating the Th17/Treg cell ratio via SCFAs presents a promising therapeutic avenue in psoriasis (Table1).

**TABLE 1:** PPARγ-mediated FAO and SCFA signaling in psoriasis immunometabolism and inflammation.

Target	Regulators	Mechanism	Effects on Treg/Th17 axis	Impact on psoriasis pathogenesis
PPARγ activation	CD36, CPT1A, AMPK	Facilitates fatty acid oxidation by upregulating fatty acid transport and mitochondrial import while concurrently suppressing glycolysis	Promotes regulatory T cell (Treg) differentiation through increased Foxp3 expression; concurrently inhibits Th17 polarization by downregulating RORγt	Leads to a reduction in IL-17 and IL-22 levels and an increase in IL-10 and TGF-β, thereby mitigating keratinocyte proliferation and psoriatic inflammation
AMPK signaling	AICAR, metformin	Stimulates FAO by activating CPT1A phosphorylation and suppresses glycolysis via mTOR inhibition	Enhances Treg lineage commitment and suppresses Th17 cell development by shifting metabolic preference toward FAO	Contributes to immune equilibrium and dampens inflammation in psoriatic lesions
SCFA–GPR axis	Butyrate, propionate, acetate	Engages G-protein coupled receptors on immune cells to mediate anti-inflammatory signaling and cellular differentiation	Facilitates thymic Treg proliferation through AIRE induction and inhibits Th17 cytokine production such as IL-17 and IL-23	Alleviates epidermal inflammation and restrains neutrophil-driven tissue damage
SCFA–HDAC inhibition	Butyrate, BA-NH-NH-BA	Inhibits histone deacetylase activity, enhancing acetylation at Foxp3 promoter regions, and represses inflammatory gene expression	Induces peripheral Treg cell development and reduces inflammatory cytokine expression through epigenetic modulation	Promotes Treg-mediated immune suppression and reduces Th17-mediated pathogenicity in psoriatic skin
Dendritic cell modulation	SCFAs (butyrate)	Suppresses dendritic cell activation, cytokine secretion, and antigen-presenting capacity via downregulation of inflammatory transcription pathways	Diminishes activation of Th17 cells by impairing dendritic cell priming functions	Decreases stimulation of the IL-23/IL-17 axis and reduces type I interferon production in lesional skin
Macrophage polarization	SCFAs, PPARγ	Promotes anti-inflammatory (M2) macrophage polarization and inhibits inflammasome-mediated cytokine maturation	Shifts macrophage phenotype toward Treg-supportive profiles and limits pro-inflammatory cytokine release	Restrains IL-1β, IL-18, and TNF-α secretion, contributing to resolution of psoriatic inflammation
Neutrophil regulation	SCFAs	Reduces neutrophil infiltration, reactive oxygen species (ROS) production, and neutrophil extracellular trap (NET) formation	Attenuates feedback amplification of Th17 responses through reduced IL-36 and chemokine signaling	Limits pustule formation and epidermal damage in psoriasis, particularly in generalized pustular subtypes

### 4.3 SCFAs attenuate neutrophil infiltration and NET-mediated inflammation in psoriasis

Neutrophilic infiltration is a hallmark of psoriatic skin, contributing to the formation of neutrophil extracellular traps (NETs) (Shao et al., 2019). In psoriasis, neutrophils release enzymes such as myeloperoxidase (MPO), neutrophil elastase (NE), proteinase 3, and cathepsin G, which generate reactive oxygen species (ROS), activate inflammatory mediators, and produce autoantigens (Rodrigues et al., 2016). Neutrophils also express numerous receptors, including Toll-like receptors (TLRs) and NOD-like receptors (NLRs), which promote the production of inflammatory cytokines (TNF-α, IL-1, IL-4, IL-6) and chemokines (CXCL1, CCL2), further aggravating psoriatic inflammation (Mantovani et al., 2011). Neutrophils in psoriatic patients exacerbate skin inflammation via NET-mediated activation of IL-36 and TLR4 signaling pathways (Shao et al., 2019). SCFAs such as butyrate and propionate suppress NETosis by inhibiting PAD4-mediated histone citrullination and chromatin decondensation, and reduce NADPH oxidase–driven ROS production via MAPK/NF-κB blockade. Additionally, SCFAs downregulate CXCR2 expression, limiting chemotactic responses to CXCL1/CXCL8 gradients. These effects involve GPR43 activation and HDAC inhibition. In murine psoriasis models, elevated SCFAs significantly reduced lesion formation, epidermal proliferation, and expression of IL-17A/F, IL-22, IL-6, TNF-α, CXCL1, and CCL20, while enhancing IL-10 and TGF-β1 levels (Chiang et al., 2019; Yoshida et al., 2023). Thus, SCFAs not only reduce neutrophil infiltration but also modulate their effector functions—including NETosis, ROS production, and chemokine-driven recruitment—thereby attenuating neutrophil-mediated immunopathology in psoriasis.

### 4.4 SCFAs in dendritic cell and macrophage -mediated modulation of psoriasis

The skin harbors a complex network of dendritic cells (DCs), including Langerhans cells (LCs), conventional dermal DCs (cDCs), plasmacytoid DCs (pDCs), and inflammatory DCs (iDCs). As primary producers of type I interferons (IFN-α), TNF-α, IL-12, and IL-23, DCs are instrumental in psoriatic pathogenesis (Wang and Bai, 2020; Macri et al., 2018). pDCs recognize viral nucleic acids and, via TLR7 and TLR9 activation, produce large quantities of IFN-α (Kim et al., 2017). IFN-α serves as an upstream trigger of the IL-23/IL-17 axis by inducing immature cDCs to secrete IL-12, IL-15, IL-18, and IL-23 (Nadeem et al., 2020; Lande and Gilliet, 2010). Additionally, antimicrobial peptides such as human β-defensins (hBD2, hBD3) and lysozyme can aggregate self-DNA or RNA into microparticles, which are phagocytosed by pDCs and activate TLR7, TLR8, and TLR9, contributing to psoriatic inflammation (Kopfnagel et al., 2018). SCFAs have been shown to attenuate the antigen-presenting capabilities of DCs, thereby reducing the activation of autoreactive T cells (Kaisar et al., 2017). SCFAs, particularly butyrate, downregulate lipopolysaccharide (LPS)-induced expression of DC activation markers and pro-inflammatory cytokine release (Nastasi et al., 2015). Notably, n-butyrate suppresses homotypic DC aggregation, inhibits IL-12 production, maintains IL-10 secretion, and blocks nuclear translocation of NF-κB at the molecular level (Säemann et al., 2002). Macrophages are pivotal regulators of immune responses and are heavily involved in psoriatic pathology. Two macrophage phenotypes exist: pro-inflammatory M1 and anti-inflammatory M2 subtypes. Psoriatic lesions are characterized by increased M1 and decreased M2 macrophage populations (Kim et al., 2019). These macrophages produce inflammatory mediators such as TNF-α. M1 macrophages also express inducible nitric oxide synthase (iNOS), IL-23p19, and IL-12/23p40, all of which are central to psoriasis pathogenesis (Fuentes-Duculan et al., 2010). SCFAs have been implicated in modulating macrophage polarization. Specifically, SCFAs promote M1-to-M2 phenotype switching and suppress the production of inflammatory mediators such as TNF-α (Wang Z. et al., 2020). Furthermore, SCFAs—especially butyrate—can inhibit the NOD-like receptor family pyrin domain containing 3 (NLRP3) inflammasome pathway, thereby preventing macrophage activation and reducing the secretion of pro-inflammatory cytokines IL-1β and IL-18 (Shao et al., 2021). These data support the notion that macrophage polarization is a key mechanism underlying the anti-inflammatory effects of SCFAs in psoriasis.

## 5 Advances in SCFA-based therapeutic strategies for psoriasis

Recent advances underscore the therapeutic potential of SCFAs, particularly butyrate, in ameliorating psoriatic inflammation through dual mechanisms: modulation of cutaneous microbiota and suppression of pro-inflammatory mediators (Nadeem et al., 2017). However, evidence from imiquimod-induced murine models of psoriasis indicates that not all SCFAs confer protective effects; notably, both topical and systemic administration of acetate exacerbated disease phenotypes (Karamani et al., 2021). These divergent outcomes underscore the context-dependent immunomodulatory properties of SCFAs, wherein distinct SCFA species may elicit either anti-inflammatory or pro-inflammatory effects depending on the local microenvironment and disease state. Despite their therapeutic promise, the clinical translation of SCFAs faces practical limitations, notably their malodorous nature and the requirement for high concentrations to achieve therapeutic efficacy. In response, structurally modified SCFA derivatives have been developed to enhance stability and bioactivity. One such derivative, BA-NH-NH-BA, consists of two butyrate moieties conjugated via an “-NH-O-NH-” linker. This compound demonstrates attenuated HDAC inhibitory activity relative to native butyrate, yet significantly suppresses *Staphylococcus aureus* proliferation and reduces inflammation in preclinical models (Traisaeng et al., 2019). Gut-derived SCFAs also exert systemic anti-inflammatory effects. For instance, *Lactobacillus* sakei extracts alleviate psoriatic manifestations in mice (Rather et al., 2018), and dietary supplementation with inulin augments microbial SCFA biosynthesis, leading to marked improvement in psoriatic dermatitis (Yoshida et al., 2023).

## 6 Conclusion

Psoriasis is sustained by a self-reinforcing loop of immune imbalance and metabolic rewiring, in which innate immune activation and lipid cues function as key amplifiers of cutaneous inflammation. This review integrates two convergent axes—PPARγ-driven FAO and SCFA signaling—to propose a unifying framework whereby lipid metabolism coordinately tunes adaptive and innate immunity to shape keratinocyte hyperproliferation and cytokine networks. Mechanistically, PPARγ enhances mitochondrial FAO through induction of CD36, CPT1, and AMPK–PGC-1α programs, thereby strengthening Foxp3 stability and IL-10/TGF-β output while restraining glycolytic–mTORC1/HIF-1α circuitry that favors RORγt-dependent Th17 polarization and IL-17/IL-22 amplification. In parallel, SCFAs signal via GPCRs (GPR41/43/109A) and epigenetic mechanisms (HDAC inhibition) to promote Treg expansion and repress Th17-associated enhancer activity, while also dampening innate effector modules, limiting NETosis, reducing ROS generation and chemotactic recruitment, and attenuating dendritic cell activation and NF-κB–dependent inflammatory transcription, collectively favoring resolution in appropriate contexts.

From a translational perspective, the fatty acid–related immune network supports an integrated “metabolism–immunity” therapeutic blueprint for psoriasis. First, targeting the PPARγ/FAO node—spanning AMPK–CPT1A control, fatty acid uptake, and mitochondrial biogenesis—offers a rational route to restore Treg fitness and immune tolerance while constraining Th17 inflammatory programs. Second, SCFAs, optimized derivatives, and microbiome-directed interventions (probiotics, prebiotics, and fiber-enriched dietary strategies) may provide scalable means to reshape immunometabolic tone and complement cytokine-targeted therapies. However, SCFA effects are context- and dose-dependent, varying by metabolite species, delivery route, ecological niche, and inflammatory stage, and thus require rigorous causal validation across models and patient strata. Future efforts should prioritize integrative multi-omics profiling for endotype stratification, single-cell and spatial validation within lesional skin, and mechanistically guided combination regimens with IL-23/IL-17 pathway blockade to achieve more durable, safe, and precision metabolic–microbiome targeting in psoriasis.
